# LACC1 bridges NOS2 and polyamine metabolism in inflammatory macrophages

**DOI:** 10.1038/s41586-022-05111-3

**Published:** 2022-08-17

**Authors:** Zheng Wei, Joonseok Oh, Richard A Flavell, Jason M. Crawford

**Affiliations:** 1. Department of Immunobiology, Yale University School of Medicine, New Haven, CT USA; 2. Institute of Biomolecular Design & Discovery, Yale University, West Haven, CT, USA; 3. Department of Chemistry, Yale University, New Haven, CT, USA; 4. Howard Hughes Medical Institute, Yale University School of Medicine, New Haven, CT, USA; 5. Department of Microbial Pathogenesis, Yale University School of Medicine, New Haven, CT, USA

## Abstract

The mammalian immune system employs various pattern recognition receptors (PRRs) to recognize invaders and host damage and transmits this information to downstream immunometabolic signaling outcomes. Laccase domain-containing 1 (LACC1) protein is an enzyme highly expressed in inflammatory macrophages and serves a central regulatory role in multiple inflammatory diseases, such as in inflammatory bowel diseases (IBDs), arthritis, and clearance of microbial infection^1-4^. However, the biochemical roles required for LACC1 functions remain largely undefined. Here, we elucidated a shared biochemical function of LACC1 in mice and humans, converting L-citrulline (L-Cit) to L-ornithine (L-Orn) and isocyanic acid (HNCO) and serving as a bridge between proinflammatory nitric oxide synthase (NOS2) and polyamine immunometabolism. We validated the genetic and mechanistic connections among NOS2, LACC1, and ornithine decarboxylase 1 (ODC1) in mouse models and bone marrow derived macrophages (BMDMs) infected by *Salmonella enterica* Typhimurium. Strikingly, LACC1 phenotypes required upstream NOS2 and downstream ODC1, and *Lacc1*^*−/−*^ chemical complementation with its product L-Orn could significantly restore wildtype activities. Our findings illuminate a previously unidentified pathway in inflammatory macrophages, explain why its deficiency may contribute to human inflammatory diseases, and suggest that L-Orn could serve as a nutraceutical to ameliorate LACC1-associated immunological dysfunctions such as arthritis or IBD.

The innate immune system is the first line of defense against infection and other injuries. It is activated by PRRs that broadly distinguish between foreign and endogenous host molecules, initiating inflammatory responses and recruiting macrophages as well as other immune cells to the site of injury^4^. In addition to a variety of essential proteins, a few small molecule families such as reactive oxygen species (ROS), reactive nitrogen species (RNS), and reactive carbonyl species (RCS) are indispensable for immune function. ROS can both promote and prevent cell death, cancer, aging and inflammation in a context-dependent manner^5,6^. RNS have been recognized to play a crucial role in the physiologic regulation of many cell types, such as smooth muscle cells, cardiomyocytes, platelets, and nervous and juxtaglomerular cells^7^. As the major source of RNS in macrophages, inducible NOS2 was originally described as an enzyme that is expressed in classically activated macrophages, generates nitric oxide (NO) from the amino acid L-arginine (L-Arg)^8^, and thereby contributes to the control of replication or killing of intracellular microbial pathogens^9^. The other product of NOS2, L-Cit, can be used as a substrate to regenerate L-Arg via argininosuccinate synthetase 1 (ASS1) and argininosuccinate lyase (ASL)^10^. However, the fate of NOS2 products remain only partially defined.

The laccase domain-containing 1 protein (LACC1) serves a central regulatory role in a variety of inflammatory diseases and is expressed predominantly in myeloid cells^1,2^. LACC1, also known as C13orf31 or FAMIN, is robustly activated by lipopolysaccharide (LPS) or poly-I:C in mouse bone marrow derived macrophages (BMDMs)^2,3^. Consistent with LACC1 activity in inflammatory macrophages, its expression is induced by macrophage colony-stimulating factor (M-CSF) in an AKT-mTOR-dependent manner during human monocyte-macrophage differentiation^11^. Frame-shift mutations and single-nucleotide polymorphisms K38E, I254 and C284R in LACC1 identified in genome-wide association studies (GWAS) are correlated with early-onset Crohn’s disease (CD), ankylosing spondylitis, systemic juvenile idiopathic arthritis (JIA), and a high-risk state for leprosy (*Mycobacterium leprae* infections)^12-17^. Mouse models deficient in LACC1 showed exacerbated arthritis, psoriasis, T cell transfer colitis (*Rag2*^*−/−*^ background), and intestinal bacterial infection (*Citrobacter rodentium* and *Salmonella enterica* serovar Typhimurium)^2,3^. Mouse LACC1 (mLACC1) deficient animals exhibited elevated proinflammatory cytokines and worsened intestinal bacterial clearance, supporting both anti-inflammatory and antibacterial functions^2,3^. Yet how such an enzyme could mediate such broad effects was unclear. Recently, human LACC1 (hLACC1) was shown to interact with a family of autophagy-associated proteins, including autophagy inducers RACK1 and AMPK, and *Lacc1* deficiency reduced autophagic flux in primary human macrophages, defining a novel form of genetically inherited JIA associated with impaired autophagy in macrophages^11^. This is consistent with enhanced expression of autophagy proteins in wildtype (WT) relative to *Lacc1*^−/−^ mouse BMDMs and partial restoration of LACC1-mediated bacterial clearance in the presence of the autophagy inducer rapamycin^3^. While LACC1 was reported to participate in several purine nucleotide metabolic reactions in mouse BMDMs^18^, these biochemical activities do not explain the autophagy phenotype. Overall, these findings support LACC1 as an important regulator of macrophage immunometabolic function^19^, but the major catalytic roles required for LACC1 function in inflammatory macrophages remained unknown.

## LACC1 is an isocyanic acid synthase

**Extended Data Fig. 1.**
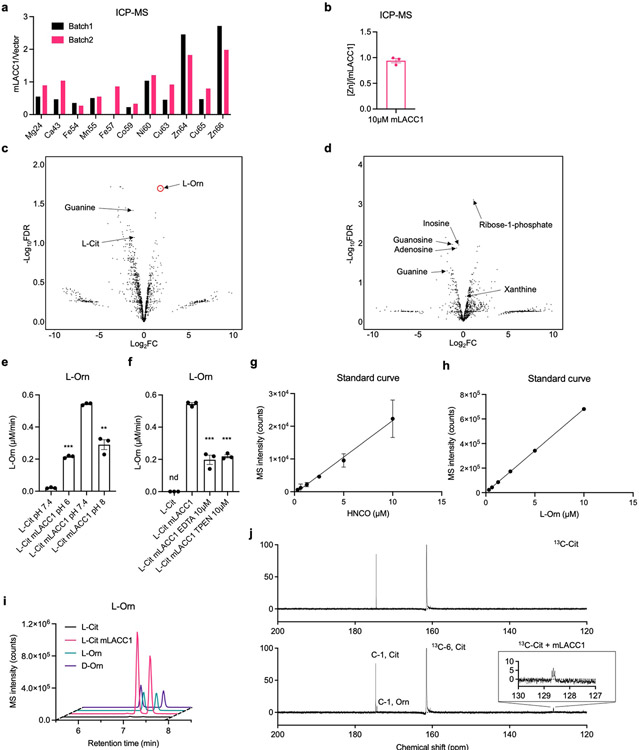
*In vitro* biochemical analysis of isolated LACC1 variants. a, ICP-MS assays showed the enrichment of Zn^64^ and Zn^66^ isotopes in recombinant mLACC1 relative to vector control. b, A quantitative ICP-MS assay for Zn showed the average Zn:mLACC1 ratio as 0.94, suggesting a 1:1 ratio in the isolated enzyme. c-d, Volcano plots from UPLC-QTOF-MS experiments showing the fold change (FC) (substrate with mLACC1 versus substrate with heat inactivated mLACC1) and false discovery rate (FDR) values collected in positive ion mode (c) and negative ion mode for the specific detection of ribose-1-phosphate (d). e, Rates of L-Orn production in enzymatic assays at different pH conditions. f, Rates of L-Orn production in enzymatic assays with the supplementation of EDTA or TPEN in the reaction buffer. g, The standard curve used for quantifying L-Orn in enzymatic assays. h, The standard curve used for quantifying 2-aminobenzoate-HNCO carbamoylation products in enzymatic assays. i, The LC-MS trace of 1-fluoro-2,4-dinitrophenyl-5-L-alanine amide (FDAA, Marfey’s reagent)-coupled L-Orn in enzymatic assays, supporting the stereoconfiguration. j, Expanded view ^13^C-NMR spectroscopic data of LACC1 reaction using 6-^13^C_1_-L-Cit as substrate, showing L-Orn and HNCO as major products. The mean and SEM (error bars) are derived from three biological replicates (n = 3). Statistical significance (two-tailed t-test) compared to control (Ctrl): *P<0.05; **P<0.01; ***P<0.001; nd, not detectable.

**Fig. 1.**
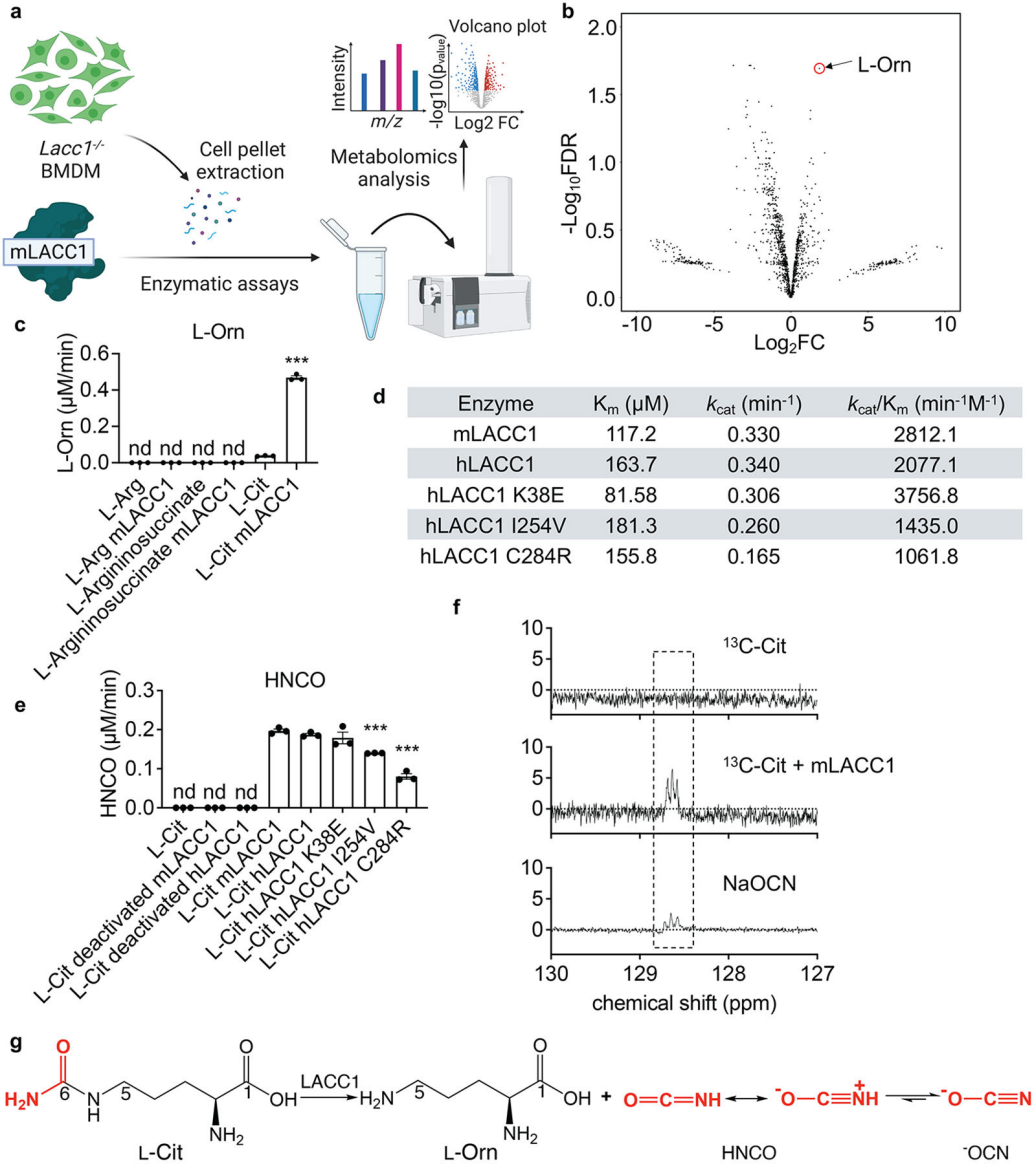
LACC1 is an endogenous isocyanic acid synthase, converting L-Cit to L-Orn and HNCO. a, Pipeline used for *in vitro* enzymatic evaluation of mLACC1. b, Volcano plot showing the fold change (FC) (substrate with mLACC1 versus substrate with heat inactivated mLACC1) and false discovery rate (FDR) values in positive ion mode. c, Abundance of L-Orn in enzymatic assays validating the substrate of mLACC1. d, Summary of kinetic parameters of mLACC1, hLACC1, hLACC1 K38E, hLACC1 I254V, and hLACC1 C284R. e, Abundance of HNCO in enzymatic assays, as assessed by the 2-aminobenzoate-HNCO carbamoylation assay. f, Direct detection of isocyanic acid (HNCO) through ^13^C-NMR spectroscopy. g, Major biochemical transformation mediated by LACC1. The mean and SEM (error bars) are derived from three biological replicates (n = 3). Statistical significance (two-tailed t-test) compared to control (Ctrl): *P<0.05; **P<0.01; ***P<0.001; nd, not detectable.

To establish the biochemical function of LACC1, we first conducted *in vitro* biochemical assays using isolated recombinant mLACC1. Mouse and human LACC1 amino acid sequences predict a multi-copper polyphenol oxidoreductase laccase-like domain (pfam02578). To first establish the metal composition of the enzyme, we analyzed isolated mLACC1 versus vector control using inductively coupled plasma mass spectrometry (ICP-MS) (Extended Data Fig. 1a-b). However, specific enrichment of Zn (0.94 metal:enzyme ratio) was observed, whereas there was no enrichment of Cu or other transition metals, suggesting that LACC1 is rather a Zn-dependent metalloenzyme. To establish a physiologically-relevant substrate mixture for LACC1, we generated a *Lacc1*^*−/−*^ mouse model and extracted the metabolites from its isolated inflammatory BMDM cells (Fig. 1a). We reasoned that the LACC1 substrates would accumulate in these cells. The metabolite extract was incubated with mLACC1 (1 h; 37°C) in phosphate buffered saline supplemented with Zn^2+^ and then analyzed by ultra-performance liquid chromatography quadrupole time-of-flight mass spectrometry (UPLC-QTOF-MS). The metabolomics data were processed and presented as a volcano plot showing fold change (FC) versus false discovery rate (FDR) values. Intriguingly, L-Orn was the most upregulated metabolite among significant hits (FDR <0.05) under these conditions (positive ion mode, Fig. 1b; negative ion mode, Extended Data Fig. 1d)^18^. L-Orn is a product of L-Arg metabolism, which is the major metabolism classification between classical and alternatively activated macrophages. In inflammatory macrophages, L-Arg is metabolized to NO and L-Cit via NOS2. By contrast, in IL-4 stimulated anti-inflammatory macrophages, L-Arg is metabolized to L-Orn and urea via arginase^20,21^.

**Extended Data Fig. 2.**
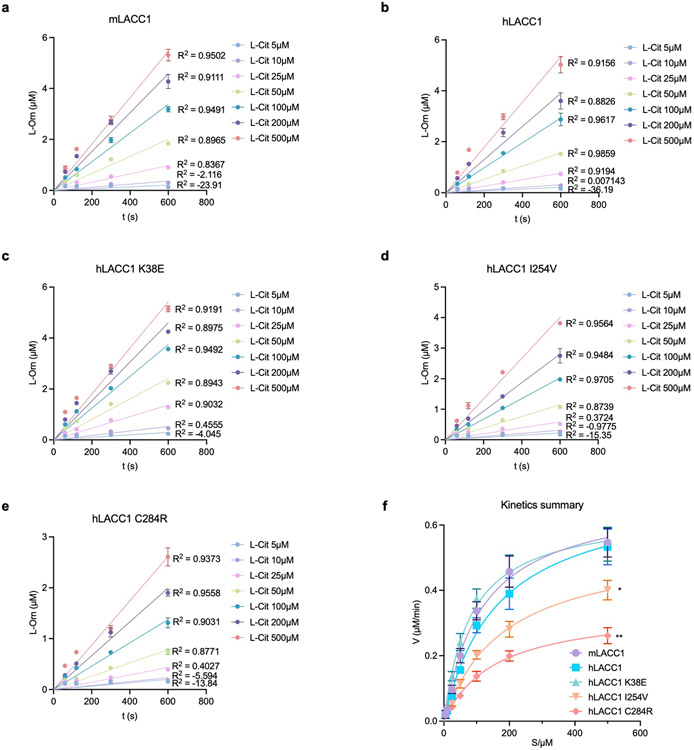
Kinetic analysis of isolated enzymes. Velocity plots of isolated enzyme reactions at different concentrations of L-Cit (5 μM, 10 μM, 25 μM, 50 μM, 100 μM, 200 μM and 500 μM) in enzyme kinetic analysis of WT mLACC1 (a) and hLACC1 (b) and the polymorphic human enzyme variants: K38E (c), I254V (d) and C284R (e). f, Michaelis-Menten curves of mLACC1, hLACC1, hLACC1 K38E, hLACC1 I254V, and hLACC1 C284R. The mean and SEM (error bars) are derived from three biological replicates (n = 3). Statistical significance (two-tailed t-test) compared to control (Ctrl): *P<0.05; **P<0.01; ns, not significant.

To validate the enzymatic transformation observed, we performed assays using L-Arg, L-argininosuccinate (L-ArgSuc), or L-Cit and found that L-Cit was the only viable substrate (Fig. 1c). The stereoconfiguration of product L-Orn was confirmed via Marfey’s analysis (Extended Data Fig. 1i). To assess the catalytic efficiencies and roles of putative hLACC1 disease polymorphisms, we also conducted enzyme kinetic analysis of isolated WT mLACC1 and hLACC1 and the polymorphic human enzyme variants: K38E, I254V and C284R. Michaelis-Menten kinetics were established using varying L-Cit substrate concentrations and measuring L-Orn product concentrations over time by liquid chromatography triple quadrupole mass spectrometry (LC-QQQ-MS) (pH 7.4, Extended Data Fig. 2a-f; pH rate profiling, Extended Data Fig. 1e). mLACC1 and hLACC1 showed comparable binding (K_m_) and catalytic turnover (*k*_cat_) values (Fig. 1d), supporting a conserved biochemical activity between mice and humans. Disease associated human polymorphisms I254V and C284R exhibited a significant reduction in turnover (*k*_cat_) and overall catalytic efficiency (*k*_cat_/K_m_), providing a catalytic mechanistic contribution to their associated immunopathologies (see below). However, disease correlative mutant K38E showed improved catalytic efficiency and binding (K_m_), which would accelerate the timing of the response in inflammatory macrophages (*i.e.*, LACC1 response would occur sooner with accumulating NOS2-derived L-Cit in this variant versus wildtype). Finally, consistent with our metal analysis, presence of metal- (ethylenediaminetetraacetic acid, EDTA) or Zn^2+^-specific (N,N,N′,N′-tetrakis (2-pyridylmethyl) ethane-1,2-diamine, TPEN) chelators in the reaction buffer significantly inhibited LACC1 activity (Extended Data Fig. 1f).

While we established that LACC1 cleaves L-Cit to L-Orn, the second fragment of the reaction could not be identified under the conditions of the studies. To directly characterize the unidentified fragment, we analyzed the mLACC1 reaction with 6-^13^C_1_-labelled L-Cit in D_2_O by nuclear magnetic resonance (NMR) spectroscopy (Fig. 1f). Strikingly, we established that LACC1 is a novel HNCO synthase, converting L-Cit to L-Orn and the RCS-like molecule HNCO (Fig. 1g), which was confirmed using a standard of its conjugate base, sodium cyanate (^−^OCN) (Fig. 1f). Only three carbon peaks were observed for this fragment, which correspond to different protonation states of the product. To detect HNCO, a carbamoylation reagent associated with uraemic syndrome^22^, by MS, we conducted 2-aminobenzoate-HNCO carbamoylation assays and measured the product by LC-QQQ-MS^23^. End-point HNCO production under saturating substrate conditions was consistent with our L-Orn kinetic measurements, including a significant reduction of activity in the human I254V and C284R mutants (Fig. 1e). Strikingly, these data suggest that LACC1 has a previously unrecognized role in L-Arg metabolism in inflammatory macrophages, bridging NOS2-derived L-Cit formation with L-Orn, a precursor in polyamine signaling, and HNCO, an RCS-like cytotoxin.

## LACC1 protects from bacterial infection

**Extended Data Fig. 3.**
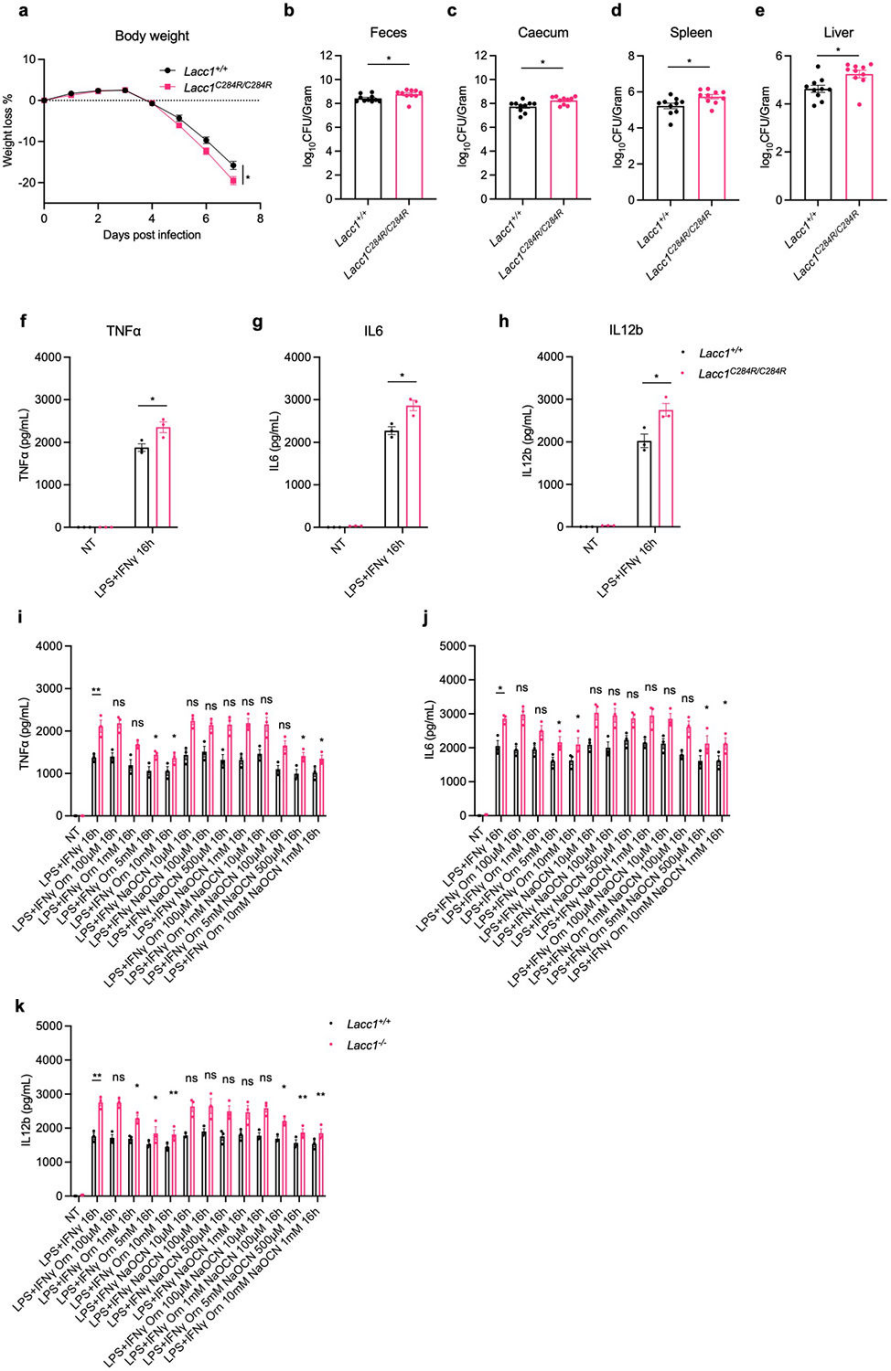
*Lacc1*^*C284R/C284R*^ shows an intermediate defect in antibacterial protection against *S*. Typhimurium infection. a, Body weight of each mouse was monitored daily after *S*. Typhimurium infection (n = 10). b, CFUs in fecal pellets were measured at day 4 after infection. c, d, e, CFUs in caecum, spleen and liver, respectively, were measured at day 6 after infection. f-h, ELISA measurements of TNFα, IL6 and IL12b secreted by BMDMs in the presence or absence (NT, non-treatment) of immunostimulant (LPS + IFNγ). i-k, ELISA measurements of TNFα, IL6 and IL12b secreted by BMDMs in the presence or absence (NT) of immunostimulant (LPS + IFNγ), exogenously supplied LACC1 product L-Orn (100 μM, 1 mM, 5 mM, 10 mM), and/or the conjugate base of LACC1 product HNCO, NaOCN (10 μM, 100 μM, 500 μM, 1 mM). The mean and SEM (error bars) are derived from three (f-k) or ten (a-e) biological replicates (n = 3 or n = 10). Statistical significance (two-tailed t-test) compared to control (Ctrl): *P<0.05; **P<0.01; ns, not significant.

**Extended Data Fig. 4.**
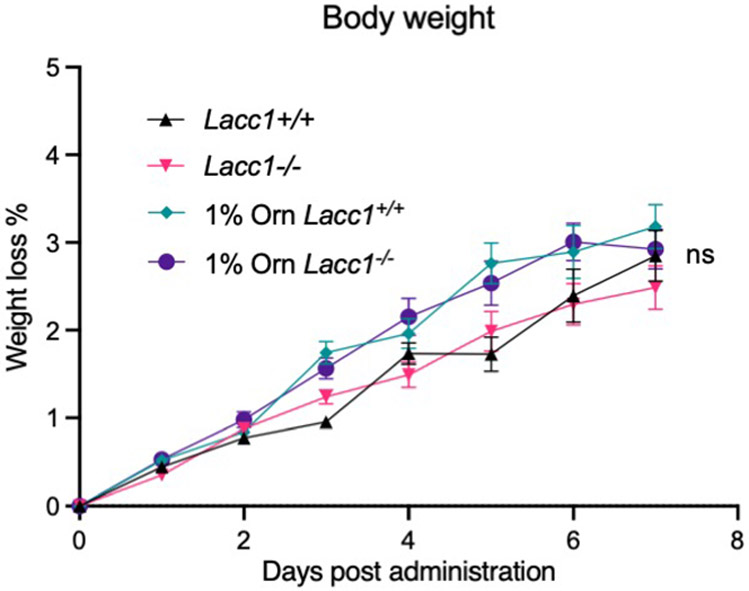
Body weight of each mouse in the absence of *S.* Typhimurium infection was monitored daily with or without 1% L-Orn administration in drinking water, showing no significant change in body weight. The mean and SEM (error bars) are derived from ten biological replicates (n = 10). Statistical significance (two-tailed t-test) compared to control (Ctrl): ns, not significant.

**Fig. 2.**
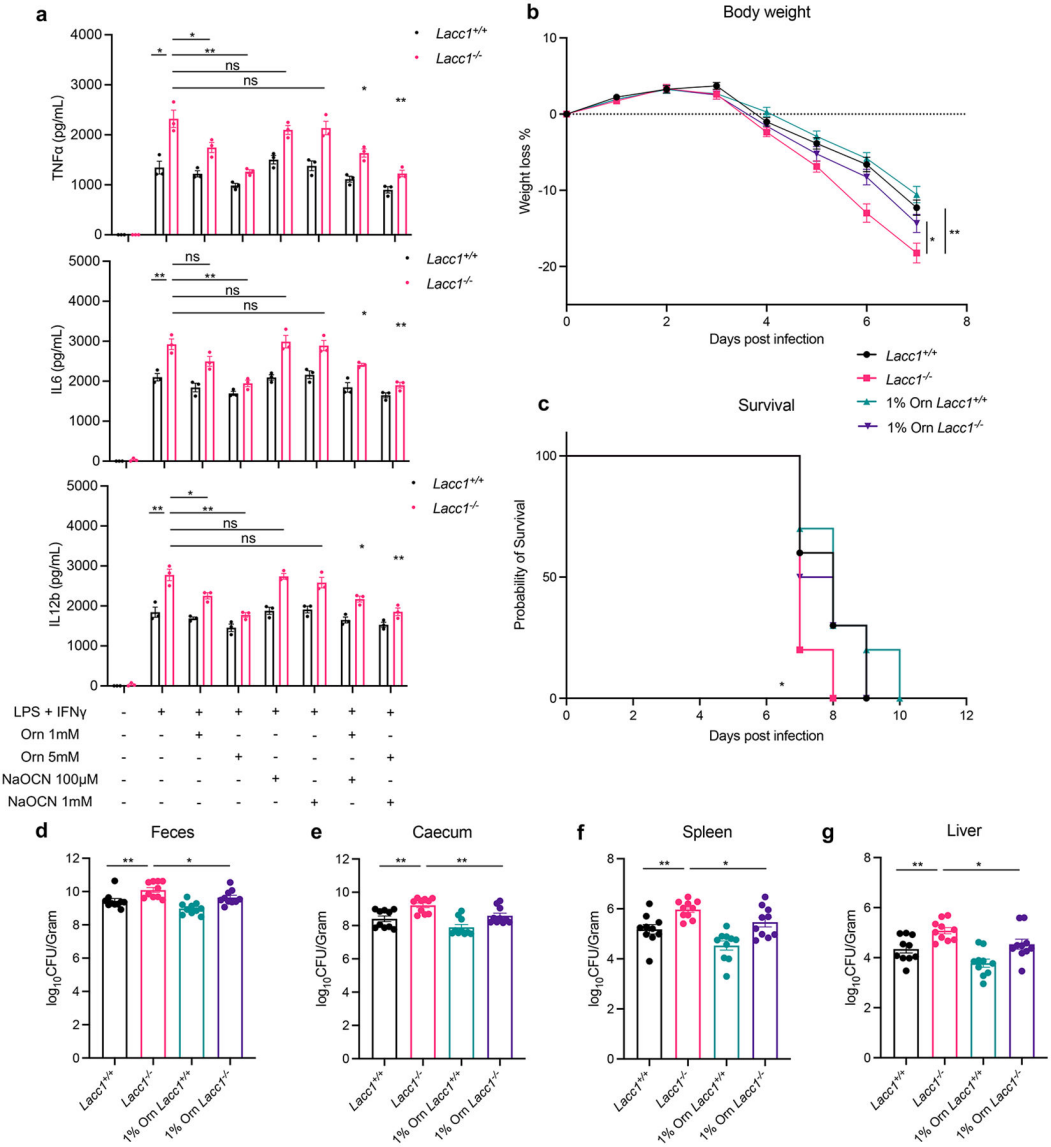
LACC1 inhibits proinflammatory cytokine signaling and protects from *S*. Typhimurium infection. a, ELISA measurements of TNFα, IL6 and IL12b secreted by BMDMs in the presence or absence of immunostimulant (LPS + IFNγ), exogenously supplied LACC1 product L-Orn (1, 5 mM), and/or the conjugate base of LACC1 product HNCO, NaOCN (100 μM, 1 mM). b, Body weight of each mouse was monitored daily after *S*. Typhimurium infection with or without 1% L-Orn administration, and the body weight loss was depicted as the percentage (%) compared to the initial body weight (n = 10). c, Mouse survival curves (n = 10). d, Colony-Forming Units (CFUs) in fecal pellets were measured at day 4 after infection. e, f, g, CFUs in caecum, spleen and liver, respectively, were measured at day 6 after infection. The mean and SEM (error bars) are derived from three (Fig. 2a) or ten (Fig. 2b-g) biological replicates (n = 3 or n = 10). Statistical significance (two-tailed t-test) compared to control (Ctrl): *P<0.05; **P<0.01; ns, not significant.

To probe the molecular mechanism of LACC1 in inflammatory BMDMs and mice, we first cultured BMDMs from WT and *Lacc1*^*−/−*^ mice and stimulated them with LPS and interferon-γ (IFNγ) for 16 hours. Key proinflammatory cytokines such as TNFα, IL6 and IL12b were upregulated in *Lacc1*^*−/−*^ BMDMs (Fig. 2a, Extended Data Fig. 3i-k), further supporting the anti-inflammatory function of LACC1. To test the function of LACC1 products on cytokine regulation, we conducted chemical complementation studies using exogenous L-Orn and HNCO in *Lacc1*^−/−^ BMDMs. Importantly, supplementation with L-Orn, but not HNCO, could significantly complement *Lacc1* cytokine suppression (Fig. 2a, Extended Data Fig. 3i-k). To validate whether L-Orn could complement *Lacc1* antibacterial deficiency *in vivo*, we performed *Salmonella* Typhimurium infection in WT and *Lacc1*^*−/−*^ mice with or without L-Orn in the drinking water. In the absence of L-Orn, the body weight loss of *Lacc1*^*−/−*^ mice was significantly higher than WT littermates (Fig. 2b). This phenotype was accompanied with worsened survival and severe bacterial burden in the feces, caecum, spleen and liver of *Lacc1*^*−/−*^ mice compared to controls (Fig. 2c-g). These data are consistent with LACC1’s antibacterial role^3,24^. Strikingly, 1% L-Orn in the drinking water significantly complemented *Lacc1* deficiency *in vivo* (Fig. 2b-g). Indeed, this oral administration of L-Orn significantly improved the effects of weight loss, survival, and bacterial burden. In the absence of *S*. Typhimurium infection, 1% L-Orn in the drinking water did not perturb the body weight of WT or *Lacc1*^*−/−*^ mice, supporting its immunological role during infection (Extended Data Fig. 4). To further validate whether the function of LACC1 depends on its enzymatic activity, we constructed *Lacc1*^*C284R/C284R*^ mice and subjected them to the same *S*. Typhimurium infection experiment. *Lacc1*^*C284R/C284R*^ mice showed an intermediate phenotype with exacerbated bacterial infection and more severe bacterial burden (Extended Data Fig. 3a-e), which was consistent with the *Lacc1*^*−/−*^ mouse studies and the C284R mutant biochemical studies. Moreover, inflammatory BMDMs from the *Lacc1*^*C284R/C284R*^ mice showed an intermediate elevation of TNFα, IL6, and IL12b upon LPS and IFNγ stimulation (Extended Data Fig. 3f-h). These data suggest that the evaluated anti-inflammatory and antibacterial functions of LACC1 are primarily mediated by L-Orn and that the human disease associated mutation reduces catalytic efficiency and confers a deficient antibacterial response *in vivo*.

## LACC1 contributes to polyamine synthesis

**Extended Data Fig. 5.**
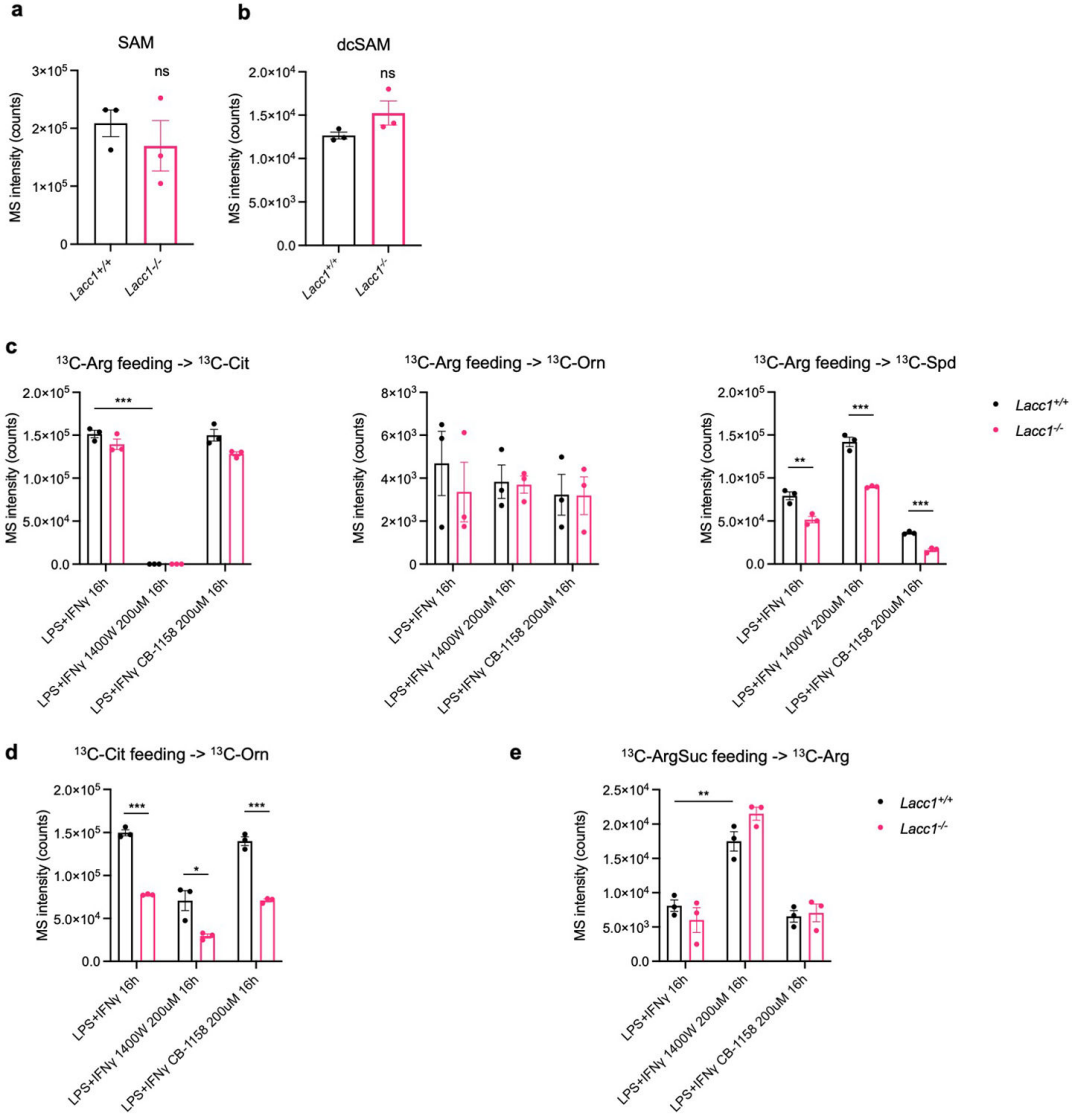
LACC1 bridges L-Arg metabolism with polyamine synthesis in inflammatory macrophages. a, b, MS intensity of SAM and dcSAM, respectively, in inflammatory BMDMs from WT and *Lacc1*^*−/−*^ mice. *Lacc1*^*−/−*^ BMDMs show an elevated but non statistically significant level of dcSAM. c-e, UPLC-QTOF-MS intensity of ^13^C-labeled metabolites in L-Arg metabolism and polyamine synthesis with ^13^C-Arg feeding (c), ^13^C-Cit feeding (d) and ^13^C-ArgSuc feeding (e), respectively. These data complement LC-QQQ-MS experimental measurements shown in Fig. 3a. The mean and SEM (error bars) are derived from three biological replicates (n = 3). Statistical significance (two-tailed t-test) compared to control (Ctrl): *P<0.05; **P<0.01; ***P<0.001; ns, not significant.

**Fig. 3.**
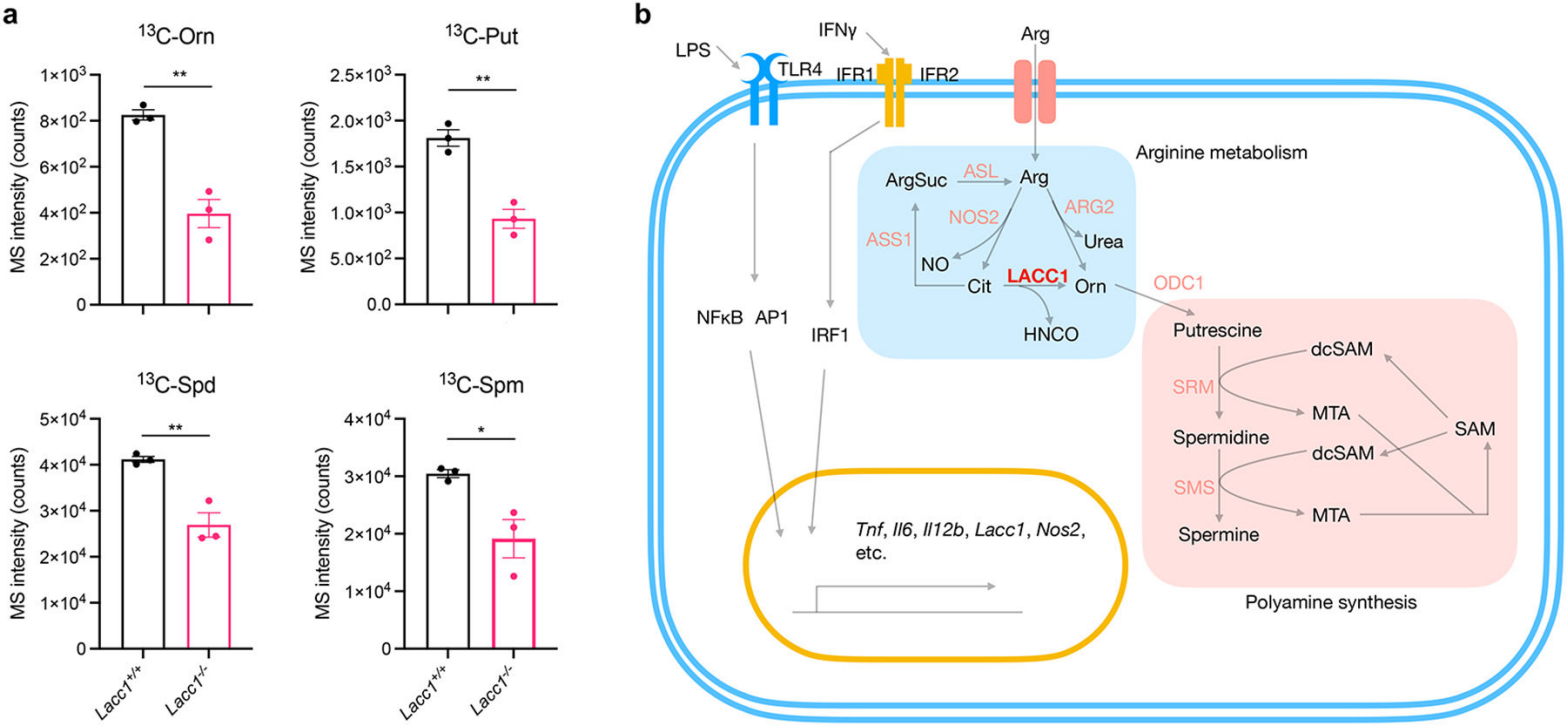
LACC1 bridges arginine metabolism with polyamine synthesis in inflammatory macrophages. a, LC-QQQ-MS intensity of ^13^C-Orn, ^13^C-Put, ^13^C-Spd and ^13^C-Spm in inflammatory BMDMs from WT and *Lacc1*^*−/−*^ mice. b, Key metabolism pathways in inflammatory BMDMs, highlighting LACC1’s novel and central role in L-Arg and polyamine metabolism. The mean and SEM (error bars) are derived from three biological replicates (n = 3). Statistical significance (two-tailed t-test) compared to control (Ctrl): *P<0.05; **P<0.01.

In macrophages, L-Orn is decarboxylated by ornithine decarboxylase 1 (ODC1) to produce putrescine for polyamine synthesis^25^. *Odc1* expression is upregulated during bacterial infections^26,27^. During polyamine synthesis, L-methionine (L-Met) is converted into S-adenosyl-L-methionine (AdoMet or SAM), which is then decarboxylated by AdoMet decarboxylase (AdoMetDC) to produce decarboxylated AdoMet (dcAdoMet or dcSAM). dcSAM is then used as an aminopropyl group donor by spermidine synthase (SRM) to convert putrescine into spermidine and spermine synthase (SMS) to convert spermidine into spermine^25^ (Extended Data Fig. 7a). To examine the impact of the enzymatic function of LACC1 on polyamine synthesis in BMDMs, we first cultured BMDMs from WT and *Lacc1*^*−/−*^ mice supplemented with 1,2,3,4,5-^13^C_5_ labeled L-Cit during differentiation. Indeed, we observed significantly reduced ^13^C-L-Orn, ^13^C-putrescine (^13^C-Put), ^13^C-spermidine (^13^C-Spd), and ^13^C-spermine (^13^C-Spm) in inflammatory BMDMs from *Lacc1*^*−/−*^ mice than those from WT mice (Fig. 3a). Additionally, there was a consistent inverse relationship with dcSAM production, but its enhancement in *Lacc1*^−/−^ BMDMs was not statistically significant (Extended Data Fig. 5a-b). Because L-Cit can also be recycled to L-Arg via ASS1 and ASL^10^ and ARG2 can directly convert L-Arg to L-Orn in IL-4 activated macrophages, we performed additional ^13^C-L-Arg, ^13^C-L-Cit, and ^13^C-L-ArgSuc isotope feeding experiments in the presence or absence of a NOS2 inhibitor, 1400W, or an ARG2 inhibitor, CB-1158 (Extended Data Fig. 5c-e). While our data support that NOS2 is the major source of L-Cit, which can indeed be recycled to L-Arg, in inflammatory macrophages as anticipated, inactivation of LACC1 significantly reduces ^13^C-L-Arg-mediated polyamine labeling regardless of inhibitor and consistent with our hypothesis. No significant LACC1 effects were observed in the ^13^C-L-ArgSuc feeding studies. These data support LACC1 as a contributor to L-Cit-mediated polyamine synthesis in inflammatory macrophages (Fig. 3b).

**Fig. 4.**
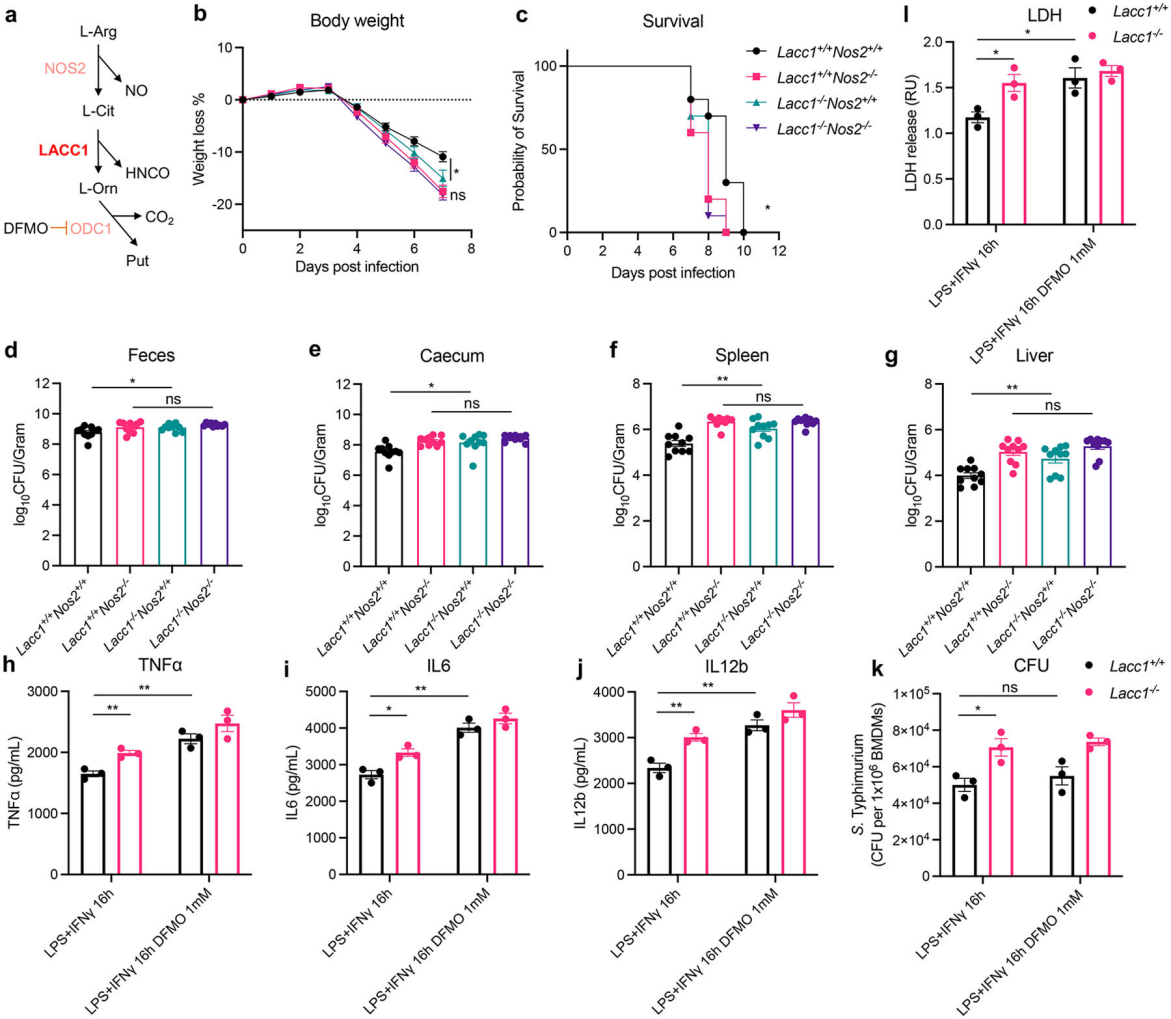
NOS2 is indispensable for the antibacterial function of LACC1. a, Illustration of the NOS2-LACC1-ODC1 signaling axis. b, Body weight of each mouse was monitored daily after *S*. Typhimurium infection (n = 10, P = 0.0414). c, Mouse survival curves (n = 10). d, CFUs in fecal pellets were measured at day 4 after infection. e, f, g, CFUs in caecum, spleen and liver, respectively, were measured at day 6 after infection. h, i, j, ELISA measurements of TNFα, IL6 and IL12b, respectively, secreted by BMDMs stimulated by LPS and IFNγ with or without 1 mM DFMO treatment. k, CFUs of intracellular *S*. Typhimurium in inflammatory BMDMs. l, LDH assay in *S*. Typhimurium infected inflammatory BMDMs. The mean and SEM (error bars) are derived from three (Fig. 4h-l) or ten (Fig. 4b-g) biological replicates (n = 3 or n = 10). Statistical significance (two-tailed t-test) compared to control (Ctrl): *P<0.05; **P<0.01; ns, not significant.

Because L-Cit is derived from NOS2 in inflammatory macrophages^8^, we proposed that LACC1 function would be dependent upon NOS2 as the latter provides the substrate for the former (Fig. 4a). To test this hypothesis, we bred *Nos2*^*−/−*^ mice with *Lacc1*^*−/−*^ mice to generate *Nos2-Lacc1* double knockout mice and measured their performance *in vivo* relative to controls in the *S*. Typhimurium infection model. We observed that NOS2 protected mice from *S*. Typhimurium infection based on body weight loss, survival, and bacterial burden in feces and invaded organs as anticipated (Fig. 4b-g), consistent with the diminished killing of *S*. Typhimurium by macrophages from *Nos2*^*−/−*^ mice^28^. However, the antibacterial function of LACC1 was abolished in *Nos2* deficient mice relative to the double knockout mice (Fig. 4b-g). Consistent with our biochemical model and BMDM results, these mouse model data suggest that the observed LACC1 function is dependent on NOS2.

**Extended Data Fig. 6.**
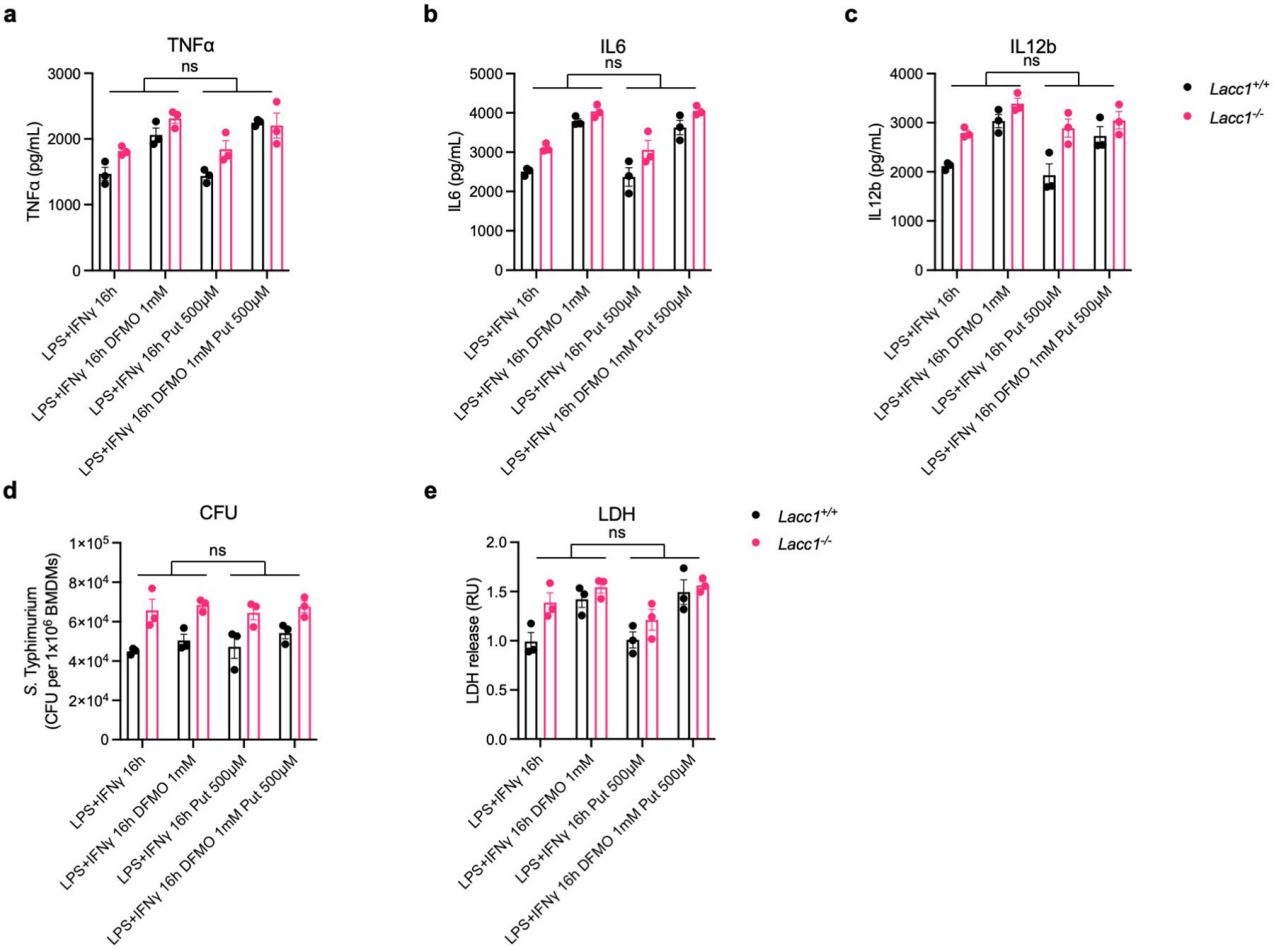
Irreversible inhibition of ODC1 phenocopied exacerbated *S*. Typhimurium induced cell death from *Lacc1*^*−/−*^ inflammatory BMDMs. a, b, c, ELISA measurements of TNFα, IL6, and IL12b, respectively, secreted by BMDMs stimulated by LPS and IFNγ with or without 1 mM DFMO and/or 500 μM putrescine (Put) treatment. d, CFUs of intracellular *S*. Typhimurium in inflammatory BMDMs. e, LDH assay in *S*. Typhimurium infected inflammatory BMDMs. The mean and SEM (error bars) are derived from three biological replicates (n = 3). Statistical significance (two-tailed t-test) compared to control (Ctrl): *P<0.05; **P<0.01; ns, not significant.

**Extended Data Fig. 7.**
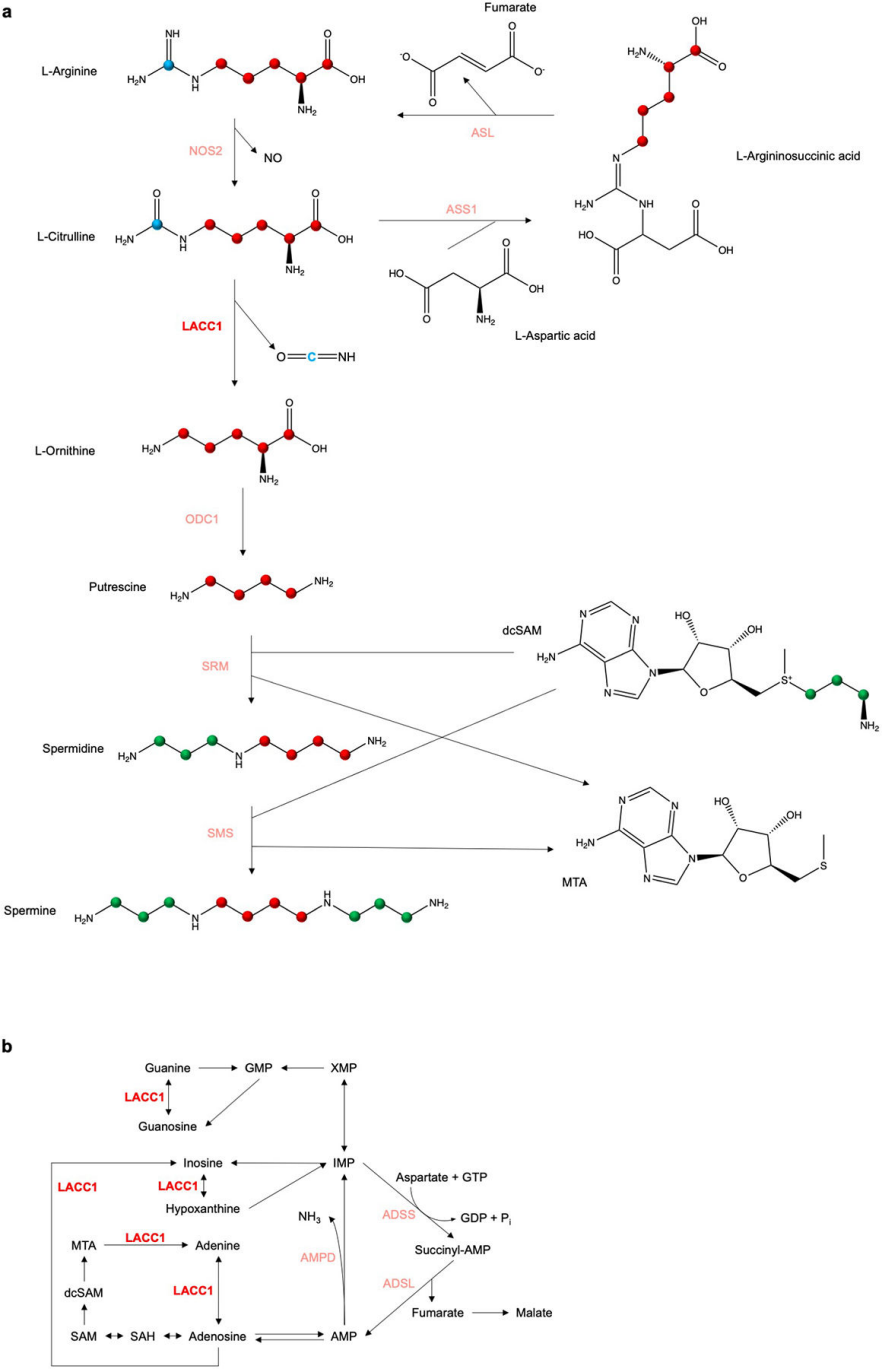
Summary of the enzymatic functions of LACC1 in inflammatory macrophages. a, LACC1’s newly described role in bridging L-Arg and polyamine metabolism in inflammatory macrophages. The carbons are highlighted with consistency to our 1,2,3,4,5-^13^C_5_-L-Cit (red) and 6-^13^C_1_-L-Cit (blue) feeding studies in inflammatory BMDMs. Carbons in spermidine and spermine highlighted in green derive from dcSAM. b, The involvement of LACC1 (a.k.a., FAMIN) in the purine nucleotide cycle as a purine nucleoside enzyme^18^. ADSL, adenylosuccinate lyase; ADSS, adenylosuccinate synthase; AMP, adenosine monophosphate; AMPD, AMP deaminase; GDP, guanosine diphosphate; GMP, guanosine monophosphate; GTP, guanosine triphosphate; IMP, inosine monophosphate; XMP, xanthosine monophosphate.

To test whether the L-Orn-polyamine axis is necessary for the anti-inflammatory and antibacterial function of LACC1, we blocked the enzymatic activity of ODC1 using the irreversible ODC1 inhibitor α-difluoromethylornithine (DFMO) in BMDMs (Fig. 4a). In contrast to cells treated with solvent vehicle, the secreted proinflammatory cytokines TNFα, IL6, and IL12b in DFMO treated BMDMs remained unchanged between WT and *Lacc1*^*−/−*^ BMDMs following LPS and IFNγ stimulation, supporting that LACC1 may suppress proinflammatory cytokine production via the downstream polyamine pathway (Fig. 4h-j). We also infected DFMO treated inflammatory BMDMs with *S*. Typhimurium. Although the normalized intracellular bacterial CFUs/macrophage remained unchanged with DFMO treatment (Fig. 4k), the lactate dehydrogenase (LDH) activity assay showed that irreversible inhibition of ODC1 phenocopied exacerbated *S*. Typhimurium induced cell death from *Lacc1*^*−/−*^ inflammatory BMDMs (Fig. 4l). This phenotype was not complemented using exogenous putrescine treatment, possibly due to dose related toxicity limiting the concentration of exogenous polyamine that could be employed (Extended Data Fig. 6e). These data suggest that intracellular LACC1-mediated polyamine synthesis protects macrophages from *S.* Typhimurium induced cell death, thereby enhancing antibacterial function. Thus, we propose a model in which LACC1 contributes to anti-inflammatory and antibacterial functions through the L-Orn-polyamine immunometabolism signaling axis (Extended Data Fig. 7a). The RCS-like cytotoxin and carbamoylation reagent HNCO^29^ likely contributes as an antimicrobial and signaling molecule, but our evaluation of activities with exogenous ^−^OCN supplementation could be masked by the overall effects of cellular ROS, RNS, and RCS in these inflammatory macrophage phenotypes.

Taken together, our study could explain how loss of *Lacc1* function in inflammatory macrophages leads to both augmented inflammatory signaling and cell death, which is consistent with both the immunopathologies and antibacterial roles associated with LACC1 in mouse models and in human diseases. This proposed inflammation equilibrium of LACC1 is analogous to other immunometabolism pathways in macrophages, such as the proinflammatory succinate- and downstream anti-inflammatory and antimicrobial itaconate signaling axis that is the result of a rewired tricarboxylic acid (TCA) cycle in inflammatory macrophages^30-33^. While other enzymatic activities of LACC1 may contribute,^18,34^ our results support a molecular mechanism in which LACC1 is a central immunometabolic regulator in L-Arg macrophage metabolism, and they may reveal the missing mechanistic connection between proinflammatory NOS2 signaling and subsequent anti-inflammatory polyamine-mediated cytokine suppression^35,36^, putative antioxidant activity^37^, and autophagy stimulation (Extended Data Fig. 7b)^38^. Indeed, polyamines such as spermidine have been reported to: 1) have a role in decreasing oxidative damage, although the mechanism is unclear^39^; 2) stimulate autophagy in mammalian cells^38^; and 3) alleviate experimental autoimmune encephalomyelitis through inducing inhibitory macrophages^40,41^. In anti-inflammatory macrophages, polyamine biosynthesis modulates mitochondrial metabolism through eIF5A hypusination, maintaining the TCA cycle and the electron transport chain (ETC) integrity^42^. In addition to supporting polyamine metabolism, LACC1 serves as an unprecedented endogenous mammalian HNCO synthase, an activity observed for antimicrobial myeloperoxidase only with specific exogenous environmental metabolites from high-fat diet and chronic exposure to cyanide, mimicking exposure to pollution and smoking^43^. While we cannot rule out other activities of LACC1-mediated L-Orn and HNCO production in cells, our molecular studies support an alternative immunometabolic proposal for LACC1 in autophagy regulation through its contribution to polyamine synthesis in addition to its reported interaction with autophagy-inducing proteins, RACK1 and AMPK^11^. These findings suggest that L-Orn could serve as a novel nutraceutical to ameliorate LACC1-associated immunological and autoimmune dysfunctions.

## Methods

### Expression and purification of LACC1.

mLACC1 and hLACC1 cDNA sequences were synthesized by GenScript, codon-optimized for prokaryotic expression, and cloned into a pMAL-C5x (New England BioLabs, N8108) vector^18^. Plasmids for human LACC1 K38E, human LACC1 I254V and human LACC1 C284R were generated using the Q5 Site-Directed Mutagenesis Kit (New England BioLabs, E0554S). *E. coli* BL21(DE3) cells were transformed with the expression plasmids and grown aerobically at 37°C and 250 revolutions per minute (rpm) in ampicillin-supplemented (100 μg/mL) Luria Bertani (LB) medium. Expression was induced at an OD_600_ of 0.5-0.6 with 0.3 mM isopropyl β-D-1-thiogalactopyranoside (IPTG) for 4 h at 30°C. Cells were harvested by centrifugation (6000 × g, 4 °C, 20 min) and cell pellets were washed twice with PBS. Cells were then resuspended in 5 mL of column buffer on ice containing 20 mM Tris-HCl, 200 mM NaCl, 1 mM EDTA, pH 7.5, and cOmplete Mini EDTA-free protease inhibitor cocktail (1 tablet per 50 mL buffer, Roche, 11873580001). The cells were lysed by sonication on ice and cleared by centrifugation at 35,000 × g for 30 minutes. The protein-containing supernatant was loaded onto a pre-equilibrated amylose resin column (New England BioLabs, E8021S). The column was washed with 20 column volumes of column buffer. The protein was eluted with 10 column volumes of column buffer supplemented with 10 mM maltose. The eluted fractions were pooled and concentrated using centrifugal filters (Millipore, UFC503096) for use in further assays.

### ICP-MS.

mLACC1 was expressed and purified as mentioned above. 1 mg purified protein was digested with 10 mL 5% HNO_3_ in a thermo digestion system, followed by the measurement of Mg, Ca, Fe, Mn, Co, Ni, Cu and Zn contents in triplicate by inductively coupled plasma mass spectrometry (ICP-MS, Perkin Elmer ICP-MS Elan DRC-e). In the averaged Zn^65^ quantification assay, 1 mL 10 μM purified mLACC1 was mixed with 10 mL 5% HNO_3_ and analyzed with Zn^65^ standards by ICP-MS.

### *In vitro* enzyme screening.

To prepare the BMDM cell pellet extract, *Lacc1*^*−/−*^ BMDMs were cultured as mentioned below and stimulated with 20 ng/mL LPS (Sigma, L4391) and 50 ng/mL IFNγ (BioLegend, 575302) for 16 hours. Cells were washed with ice-cold PBS twice and extracted with ice-cold MeOH. The extraction mixture was centrifuged and the supernatant was dried using N_2_ gas flow. 1 mg *Lacc1*^*−/−*^ BMDM cell pellet extract was incubated with 10 μM mLACC1 in 100 μL PBS buffer supplemented with 1 μM ZnSO_4_ in triplicate for 1 h at 37°C and quenched by 200 μL ice-cold acetonitrile (AcCN). The reaction mixture was centrifuged at 20,000 × g for 10 min and the supernatant was analyzed using an Agilent iFunnel 6550 quadrupole time of flight (Q-TOF) MS instrument fitted with an electrospray ionization (ESI) source coupled to an Agilent 1290 Infinity HPLC system with an Xbridge BEH Amide XP HILIC 2.5 μm, 2.1 mm x 100 mm column (Waters, 186006091). The column was maintained at 25°C during analysis. The mobile phase A was 20 mM ammonium acetate/0.1% formic acid pH 3.5. The mobile phase B was 100% acetonitrile. The flow rate was 0.22 mL/min. The gradient elution was as follows: 0 min: 85% B; 0.5 min: 85% B; 9 min: 35% B; 11 min: 2% B; 12 min: 85% B; 25 min: 85% B. The data were acquired in positive and negative ion mode (Extended Data Fig. 1d) with a full scan range of 50-1700 *m/z*, respectively. The metabolomics data were processed with Mass Profiler Professional Software 14.0 (Agilent) and presented as a volcano plot showing fold change (FC) versus false discovery rate (FDR) values. Fig. 1a was created with BioRender.

### Measurement of HNCO.

For the direct measurement of HNCO through NMR, 6-^13^C_1_-labelled L-Cit (1 mg) was incubated with 10 μM mLACC1 in D_2_O (200 μL) for 1 hour and the reaction was quenched in liquid N_2_. The reaction mixture was then subjected to ^13^C NMR to detect cyanate and its conjugate acid HNCO with reference to its central ^13^C chemical shift value of 128.64 ppm. The negative control was prepared as described above without mLACC1, and the chemical standard used was 1 mM NaOCN in D_2_O, which showed identical ^13^C chemical shift values as the enzyme product. Some minor spontaneous carboxylate carbamoylation of substrate L-Cit and product L-Orn was observed over extended incubation times (not shown). The measurement was performed using an Agilent 800 MHz NMR instrument equipped with a ^13^C-sensitivity enhanced salt-tolerant cold probe. For the indirect measurement of HNCO, an HNCO carbamoylation assay was used^23^. The enzymatic assay was performed as mentioned above and quenched with 200 μL MeOH. The reaction mixture was incubated with 20 mM acetic acid and 50 mM 2-aminobenzoic acid at 42°C for 30 min. Then, the mixture was centrifuged and the supernatant was analyzed by UPLC-QTOF-MS (Phenomenex Kinetex C18 (100 Å) 5 μm (250 × 4.6 mm^2^) column; flow rate, 0.7 mL/min; mobile phase composition, 10-100% acetonitrile in water containing 0.1% formic acid for 30 min).

### Measurement of L-Orn.

The enzymatic assay was performed as mentioned above. In some conditions as noted, KH_2_PO_4_ or Na_2_HPO_4_ was used for pH adjustment while 10 μM EDTA or 10 μM TPEN was added for metal chelation. The reaction mixture was quenched with 200 μL MeOH. Then, the mixture was centrifuged and coupled with 1-fluoro-2,4-dinitrophenyl-5-L-alanine amide, FDAA (Thermo, 48895) per the manufacturer’s protocol. The samples were analyzed by UPLC-QTOF-MS (Phenomenex Kinetex C18 (100 Å) 5 μm (250 × 4.6 mm^2^) column; flow rate, 0.7 mL/min; mobile phase composition, 10-100% acetonitrile in water containing 0.1% formic acid for 30 min).

### Measurement of enzymatic parameters.

The enzymatic assay was performed in triplicate as mentioned above at different concentrations of L-Cit (5 μM, 10 μM, 25 μM, 50 μM, 100 μM, 200 μM and 500 μM) over time (60 s, 120 s, 300 s, and 600 s). The velocity was calculated, and the Michaelis-Menten curves were plotted for the measurement of K_m_ and *k*_cat_ using Prism 9 (GraphPad).

### Mouse studies.

WT C57BL/6, *Lacc1*^*−/−*^, *Lacc1*^*C284R/C284R*^ and *Nos2*^*−/−*^ mice were used in this study. *Nos2*^*−/−*^ mice (002609-B6.129P2-*Nos2*^*tm1Lau*^/J, Jackson Laboratory) were a gift from Dr. Victor Laubach^44^. *Lacc1*^*−/−*^ mice were generated with CRISPR-Cas9 technology into C57/B6N embryos as previously described^45^. To generate *Lacc1*^*−/−*^ mice, two sgRNAs (AAACTGCCATGAGACCTTAC**TGG**, GTTAAGGCCATCGCGGACAT**GGG**) were used collectively to target exon 3 and exon 7, respectively. To generate *Lacc1*^*C284R/C284R*^ mice, an sgRNA (GAAGACGATGGGTATACAGT**CGG**) and an oligo donor (CCTACCGGAGTGAGCAACCCCACATGCCTTTTTCACAGGATCTGCGAAGACGATGGGTATACgGTCGGCGCCAAGAGCAGTGATTGTGACTCCTCTCTGAT TTGTGACGATCCCATCGTAAGA) were used collectively for homologous recombination. Experimental littermates were generated from heterozygote × heterozygote breeding. Sample size (n = 10) was chosen in line with pervious experimental experience and consistent with the broader literature^46^. 8-10-week-old male and female mice were used in equal quantities unless otherwise specified. All experiments were performed using co-housed mouse littermate controls. Isosexual male or female littermates were co-housed at 21-24°C and 40-60% humidity. A 12/12 h light/dark cycle was used. All mouse studies were performed in compliance with Yale Institutional Animal Care and Use Committee protocols. No formal blinding or randomization was conducted; however, control and treated groups were chosen arbitrarily for each experiment. Mouse weights and CFUs were measured in a blinded manner.

### Cell culture studies.

BMDMs were generated from progenitor cells isolated from femurs and tibias of mice and maintained in DMEM medium (Gibco, 11965118) with 10% FBS (Sigma, F8192-500Ml), 1% penicillin/streptomycin (Gibco, 15070063) and 50 ng/mL M-CSF (R&D, 416-ML-010) for 6 days. Cells were reseeded and stimulated with 20 ng/mL LPS (Sigma, L4391) and 50 ng/mL IFNγ (BioLegend, 575302) in triplicate for 16 hours. In some experiments as indicated, 1 mM DFMO (Cayman, 16889) was supplemented during differentiation to inhibit ODC1. After a 16-hour stimulation period, culture supernatants were collected for TNFα, IL6 and IL12b ELISA assays (R&D, DY410-05, DY406-05, DY499-05) according to the manufacturer’s protocols.

### *S*. Typhimurium infection *in vivo*.

As previously described^47^, before infection, 8-10-week-old mice (n = 10) were restricted from food and water for 4 h followed by gavage of 20 mg streptomycin. After 20 h, mice were fasted again for 4 h and infected with streptomycin resistant *S*. Typhimurium (SL1344 strain, provided by J. Galan). *S*. Typhimurium was maintained as a glycerol stock at −80°C. Before infection, bacteria were propagated overnight (37°C, 250 rpm) in LB supplemented with 100 μg/ml streptomycin. The bacterial culture was subcultured for 4 h the next day in antibiotic-free LB supplemented with 0.3 M NaCl to return it to log phase growth and increase virulence^48^. Using spectrophotometry, bacterial CFUs were calculated with an infection dose of 1 × 10^3^ CFUs per mouse. To calculate faecal CFUs, faecal pellets were resuspended in PBS at 50 mg/mL and vortexed for 20 min. Bacteria containing supernatants were clarified by centrifugation at 50 × g for 10 min. Serial dilutions were conducted, and bacteria were plated in triplicate on LB streptomycin (100 μg/mL) plates. For caecal, liver and spleen CFU enumeration, organs were isolated, weighed, and added to 2 ml of PBS. Tissue was dissociated with GentleMacs C Tubes (Miltenyi Biotech) per the manufacturer’s instructions. CFUs were calculated using similar methodology as above.

### *S*. Typhimurium infection in BMDMs.

After a 16-hour stimulation period as mentioned above, the BMDM culture medium was replaced with antibiotic-free medium. BMDMs (1 × 10^6^) were infected in triplicate with 1 × 10^7^ CFUs of *S*. Typhimurium (SL1344 strain, provided by J. Galan, MOI = 10) and centrifugated at 800 × g at 37 °C for 10 min and returned to the incubator for an additional 20 min. Medium was then removed and replaced with medium containing 100 μg/mL gentamycin to kill extracellular bacteria for 1 h (*i.e.*, the gentamycin protection assay^47^). Medium was replaced with fresh medium including 25 μg/mL gentamycin. An hour later, supernatants were sampled for LDH activity with the CyQUANT™ LDH Cytotoxicity Assay (Thermo, C20300) according to the manufacturer’s protocol. After an additional 3.5-hour culture period in the incubator, cells were washed twice with PBS and lysed in a 1% Triton and 0.1% SDS buffer for 5 min under gentle agitation. Cell lysates were plated on streptomycin (100 μg/mL) containing LB plates and incubated overnight at 37°C, and then CFUs were enumerated.

### ^13^C-isotope labelling studies.

BMDMs were generated as mentioned above, reseeded at a density of 1×10^6^ cells/mL in phenol red-free DMEM medium (Gibco, 21063029) with 10% FBS (Sigma, F8192-500Ml), 1% penicillin/streptomycin (Gibco, 15070063), and 1 mM 1,2,3,4,5,6-^13^C_6_ labeled L-Arg (Cambridge Isotope Laboratories, CLM-2265-H-0.05), 2 mM 1,2,3,4,5-^13^C_5_ labeled L-Cit (Cambridge Isotope Laboratories, CLM-8653-PK), or 1 μM 1,2,3,4,5,6-^13^C_6_, ^15^N_4_ labeled L-ArgSuc (Cambridge Isotope Laboratories, CNLM-9007-CA-0.1MG) and stimulated in triplicate with 20 ng/mL LPS (Sigma, L4391) and 50 ng/mL IFNγ (BioLegend, 575302) for 16 hours. The cells were washed twice with 10 mL ice-cold PBS, scraped in 1 mL ice-cold PBS, transferred to 1.5 mL tubes and pelleted (1 min, 6,000 × g, 4°C). The cells were washed once with 500 μL ice-cold PBS. Cellular metabolites were extracted with 50 μL of ice-cold extraction solvent (40:40:20 vol/vol/vol acetonitrile:methanol:water, 0.5% formic acid). After a 5-min incubation on ice, the acid was neutralized using NH_4_HCO_3_. After centrifugation (15 min, 20,000 × g, 4°C), the clarified supernatant was transferred to an LC-MS vial and analyzed by an Agilent iFunnel 6550 QTOF-MS instrument fitted with an ESI source or an Agilent 6490 ESI-QQQ-MS/MS instrument coupled to an Agilent 1290 Infinity HPLC system with an Xbridge BEH Amide XP HILIC 2.5 μm, 2.1 mm x 100 mm column (Waters, 186006091). The column was maintained at 25°C during the analysis. The mobile phase A was 20 mM ammonium acetate/0.1% formic acid pH 3.5. The mobile phase B was 100% acetonitrile. The flow rate was 0.4 mL/min. The gradient elution was as follows: 0 min: 95% B; 0.5 min: 95% B; 3 min: 70% B; 6 min: 40% B; 6.5 min: 0% B; 9.5 min: 0% B; 10 min: 95% B; 15 min: 95% B. MassHunter Qualitative Analysis B.07.00 (Agilent) was used to perform peak picking, peak alignment, and peak intensity integration.

## Extended Data

## Data Availability

Supplementary information and source data are provided within this letter. Additional data that support the findings of this study are available from the corresponding author upon reasonable request. Code was not used in this study.
